# Comparative genomics of *Giardia duodenalis* sub-assemblage AI beaver (Be-2) and human (WB-C6) strains show remarkable homozygosity, sequence similarity, and conservation of VSP genes

**DOI:** 10.1038/s41598-024-63783-5

**Published:** 2024-06-12

**Authors:** Rodrigo de Paula Baptista, Matthew S. Tucker, Matthew J. Valente, Subodh K. Srivastava, Nadya Chehab, Alison Li, Jahangheer S. Shaik, Juan David Ramirez, Benjamin M. Rosenthal, Asis Khan

**Affiliations:** 1 Houston Methodist Research Institute, Houston, TX 77030 USA; 2 Department of Medicine, Weill Cornell Medicine College, New York, NY 10065 USA; 3 Animal Parasitic Diseases Laboratory, Agricultural Research Service, US Department of Agriculture, Beltsville, MD 20705 USA; 4 Insights and Analytics, Applied Data Science and Learning, Data Science Institute, Takeda, Cambridge, MA 02142 USA; 5 Centro de Investigaciones en Microbiología y Biotecnología-UR (CIMBIUR), Facultad de Ciencias Naturales, Universidad del Rosario, Bogotá, Colombia; 6 Molecular Microbiology Laboratory, Department of Pathology, Molecular and Cell-Based Medicine, Icahn School of Medicine at Mount Sinai, New York, NY 10029 USA

**Keywords:** Long-read sequencing, Genome assembly, *Giardia*, Annotation, Ploidy, Synteny, Evolutionary genetics, Phylogenetics, Population genetics, Microbial genetics, Parasitology, Evolution, Microbiology

## Abstract

*Giardia duodenalis*, a major cause of waterborne infection, infects a wide range of mammalian hosts and is subdivided into eight genetically well-defined assemblages named A through H. However, fragmented genomes and a lack of comparative analysis within and between the assemblages render unclear the molecular mechanisms controlling host specificity and differential disease outcomes. To address this, we generated a near-complete de novo genome of AI assemblage using the Oxford Nanopore platform by sequencing the Be-2 genome. We generated 148,144 long-reads with quality scores of > 7. The final genome assembly consists of only nine contigs with an N50 of 3,045,186 bp. This assembly agrees closely with the assembly of another strain in the AI assemblage (WB-C6). However, a critical difference is that a region previously placed in the five-prime region of Chr5 belongs to Chr4 of Be-2. We find a high degree of conservation in the ploidy, homozygosity, and the presence of cysteine-rich variant-specific surface proteins (VSPs) within the AI assemblage. Our assembly provides a nearly complete genome of a member of the AI assemblage of *G. duodenalis,* aiding population genomic studies capable of elucidating *Giardia* transmission, host range, and pathogenicity.

## Introduction

The etiological agent of giardiasis, *Giardia duodenalis* causes widespread diarrheal disease^1,2^. More than 280 million cases of human infections are reported annually^3,4^. Giardiasis causes nausea, vomiting, diarrhea, and impaired growth and cognitive development^5^. Asymptomatic infections also occur^6–8^. *Giardia* infection causes malnutrition and impairs cognitive development routinely in children in developing countries, where young children commonly contract infection^9^. Species of *Giardia* also infect wildlife and domestic animals, such as livestock, dogs, and cats^10–13^.

Current nomenclature recognizes distinctions among *G. duodenalis* (infecting mammals and birds), *G. agilis* (amphibians), *G. muris* (rodents), *G. microti* (rodents), *G. psittaci* (psittacine birds), *G. cricetidarum* (hamsters), *G. peramelis* (quenda) and *G. ardeae* (herons)^14–17^. Within *G. duodenalis,* eight genetic assemblages (A through H) differentiate among taxa; host specificity, and genetic differences support these distinctions. Among these eight assemblages, A and B are primarily found in humans; other assemblages, such as C, D, E, and F, occur in dogs, cats, and wild canids^10^.

Antigenic and genetic differences support further subdivision of assemblage A into AI, AII, and AIII^13,18–20^. While the AI assemblage is mainly zoonotic, the most transmission of AII occurs among people^17,21^. Recently, Seabolt et al.^22^ proposed recognizing sub-assemblage AII as a separate species, *G. hominis*, preserving the name *G. duodenalis* for sub-assemblage AI, citing host specificity and evidence of gene content and population genetic structure. Although most human infections involve assemblages AII and B^7^, the molecular mechanisms controlling differences in the host range remain largely undefined^7^. Further defining and comparing parasite genomes might elucidate what governs host specificity.

*Giardia* trophozoite cell harbors two diploid, nearly identical, and functionally equivalent nuclei that are inherited independently during mitosis, located anteriorly with respect to the long axis^14^. Each nucleus contains five monocentric chromosome pairs^23–25^. The genome size varies between 11 and 13 Mb. The ploidy of *G. duodenalis* nuclei and cells varies between 2 and 16N among different life cycle stages, with trophozoites having 4N and 8N, and encysting cells ranging from 8 to 16N^26^.

*A*s few as 10 cysts are sufficient to establish an infection in humans. Newly excysted cells called excyzoites^26^, harbor four nuclei each, which divide to form four trophozoites, each containing 2N nuclei. During mitosis, the trophozoite contains two separate and independent nuclei that are physically and genetically distinct. Each nucleus is segregated into daughter cells by two individual spindles^27^. *Giardia* has not been reported to undergo mating or meiosis, although, the presence of distinct patterns of genetic variation between lineages supports genetic exchange^28^. If *Giardia* reproduces asexually exclusively, it should accumulate significant allelic heterozygosity within and between the two nuclei^23,28^. Against expectations, nuclei do not accumulate appreciable numbers of distinct mutations and heterozygosity in *G. duodenalis* appears less than 0.1% for isolates in the A1 subassemblage^22,25,29,30^; the mechanisms maintaining reduced heterozygosity remain poorly understood.

GenBank presently harbors 30 whole genome assemblies for *G. duodenalis* (https://www.ncbi.nlm.nih.gov/genbank/). The current reference (WB-C6, a clone from the original isolate WB, was isolated from a 29-year-old male in Afghanistan^31,32^) has recently been re-sequenced using PacBio long-read technology and optical mapping^24^. That assembly encompasses 35 contigs scaffolded into chromosomes, indicating the presence of remaining gaps. Other available genome assemblies are even more fragmented, encompassing more than 100 pieces each^30^. Such fragmentation impairs the understanding of genomic structural variation and genome evolution at the chromosomal level. This fragmentation resulted from the use of short-read sequencing technologies, such as Illumina (100–300 bp read length), which provides excellent base call accuracy (valuable for defining single nucleotide polymorphisms) but limits assembly contiguity mainly in repetitive and low complexity regions.

The use of third-generation sequencing has revolutionized the field of genomics by providing much longer reads, averaging around 10–20 kb. Although these longer reads are more prone to base-calling errors, they can help resolve the assembly of complex regions, resulting in a more contiguous genome assembly. Combining long-read and short-read technologies can produce an accurate, contiguous assembly. Single-molecule long-read sequencing using Oxford Nanopore Technologies (ONT) has been used to generate de novo assemblies for *Toxoplasma*^33^, *Plasmodium*^34^, and *Giardia* parasites^35^. Hence, whole genome sequencing employing long-read sequence data will enable comparative genomics within the diplomonads lineages to understand the pattern of heterozygosity and structural variant detection, such as duplications, translocations, and inversions^33,35^.

A complete reference genome that accurately represents structural variation will advance the goal of understanding the evolutionary history, population genetic structure of *Giardia,* and the remarkable sequence similarity of the two nuclei within a cell. It is worth noting that sub-assemblage AI is zoonotic, and a human isolate (WB-C6) has been assembled to the chromosomal level using PacBio long-read^24^. Here, we generated a chromosomal level genome assembly of a beaver isolate Be-2^28^ and compared it to two other isolates in the AI sub-assemblage of *G. duodenalis*: WB-C6 (human isolate)^24^ and Be-2 (beaver isolate)^28^. We sought to understand the genetic diversity, homozygosity, and conservation of virulence gene targets for these parasites by including other isolates subjected to whole genome sequencing using long-read technology.

## Results

### Genome assembly of *Giardia* Be-2

**Figure 1:**
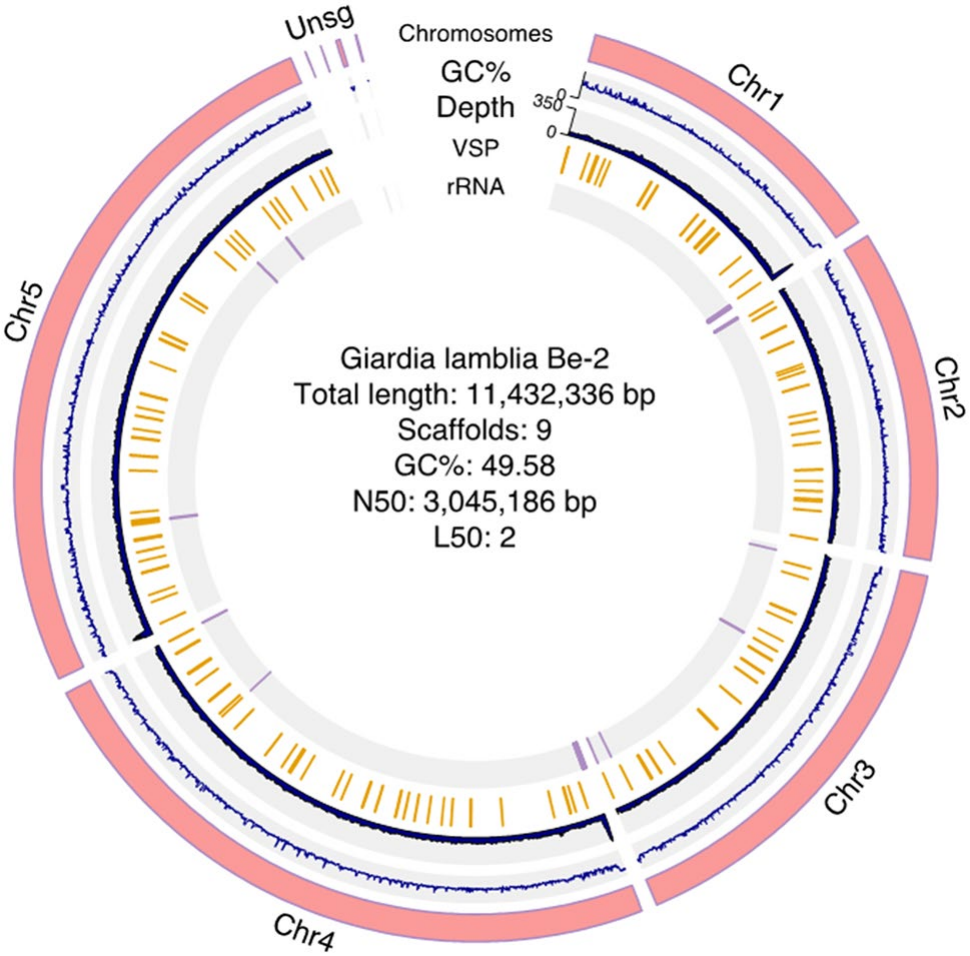
Composition of the *G. duodenalis* AI Genotype. Circos representation of the five chromosomes of *G. duodenalis* AI genotype based on strain Be-2. The outermost track indicates the chromosomal sequences. UNSG represents the 4 unassigned scaffolds containing 24,631 bp. Inner tracks show GC content and chromosomal position of variant-specific surface proteins (VSPs) and tRNAs. The annotation was made in two steps using Liftoff v.1.6.3^56^ and AUGUSTUS^57^ as mentioned in the Method section. The Circos plot was drawn with R library circlize^55^.

To generate a de novo assembled genome of *G. duodenalis* AI assemblage and to conduct a comparative genomics analysis with others in assemblage AI, we used a single flow cell Oxford Nanopore Technologies, generating 148,144 long reads with quality scores of > 7 (mean read length 5786 bp, mead read quality 11.2, read length N50 12,297.0). We processed these reads for further quality assessment to establish optimal assembly parameters. We improved the assembly by using reads longer than 1,000 bases overlapping other reads by at least 500 bases and high corMhap sensitivity. After quality assessment 105,034 reads passed QC, achieving an average coverage of 84.18-fold De novo assembly of high-quality reads produced 9 contigs with an N50 of 3,045,186 bp (Table 1, Fig. 1). The five largest contigs represent the five chromosomes of *Giardia;* they collectively span 11,432,336 bp with only one internal gap; this compares favorably with the 4 contigs, including 137 gaps, in the assembly of the reference WB-C6 strain^23,24^ (Table 1, Fig. 1). Our assembly is larger than the published A2 lineage genome of DH_A2 (10,703,894 bp), which is fragmented into 239 contigs^36^.

**Table 1:** Genome assembly statistics.

Assembly	*G. duodenalis* Be-2*	*G. intestinalis* WB-C6^24^**	*G. intestinalis* WB^35^	*G. intestinalis* GS^35^	*G. intestinalis* Beaver^35^
Contigs (≥ 0 bp)	9	35	37	19	8
Total length (≥ 0 bp)	11,432,336	12,078,186	11,696,115	13,164,248	11,467,485
Largest contig	3,341,382	4,018,654	1,573,360	2,326,275	2,759,360
GC (%)	49.58	49.73	49.52	49.19	49.56
N50	3,045,186	2,761,001	616,181	1,645,020	1,964,742
L50	2	2	7	4	3
# N's per 100 kbp	0.87	3,350.76	0	0	0

*Current assembly of *G. duodenalis* Be-2.

**To obtain consistent results, we reanalyzed the WB genomes for GC (%) content using GCcalc.py (https://github.com/WenchaoLin/GCcalc) with 1 kb sliding window size.

### Genome assembly at the chromosomal level resolves gapped regions in the current WB-C6 reference genome

To gain a better understanding of genomic variation within *G. duodenalis* AI sub-assemblage, we conducted a comparison of the genome assembly of *G. duodenalis* Be-2 and WB-C6. The new genome assembly of Be-2, resulted in a smaller, more contiguous genome than the WB-C6 reference (12.6 Mbp)^24^ (Table 1, Fig. 1). The only gap found in the new Be-2 assembly occurred in Chr3, totaling just 100N bases close to the three-prime end region of this chromosome (Table 1), which contains sub-telomeric content. This junction is highly syntenic to the WB-C6 genome and is also supported by the presence of a few long-reads to connect with the surrounding contigs.

**Figure 2:**
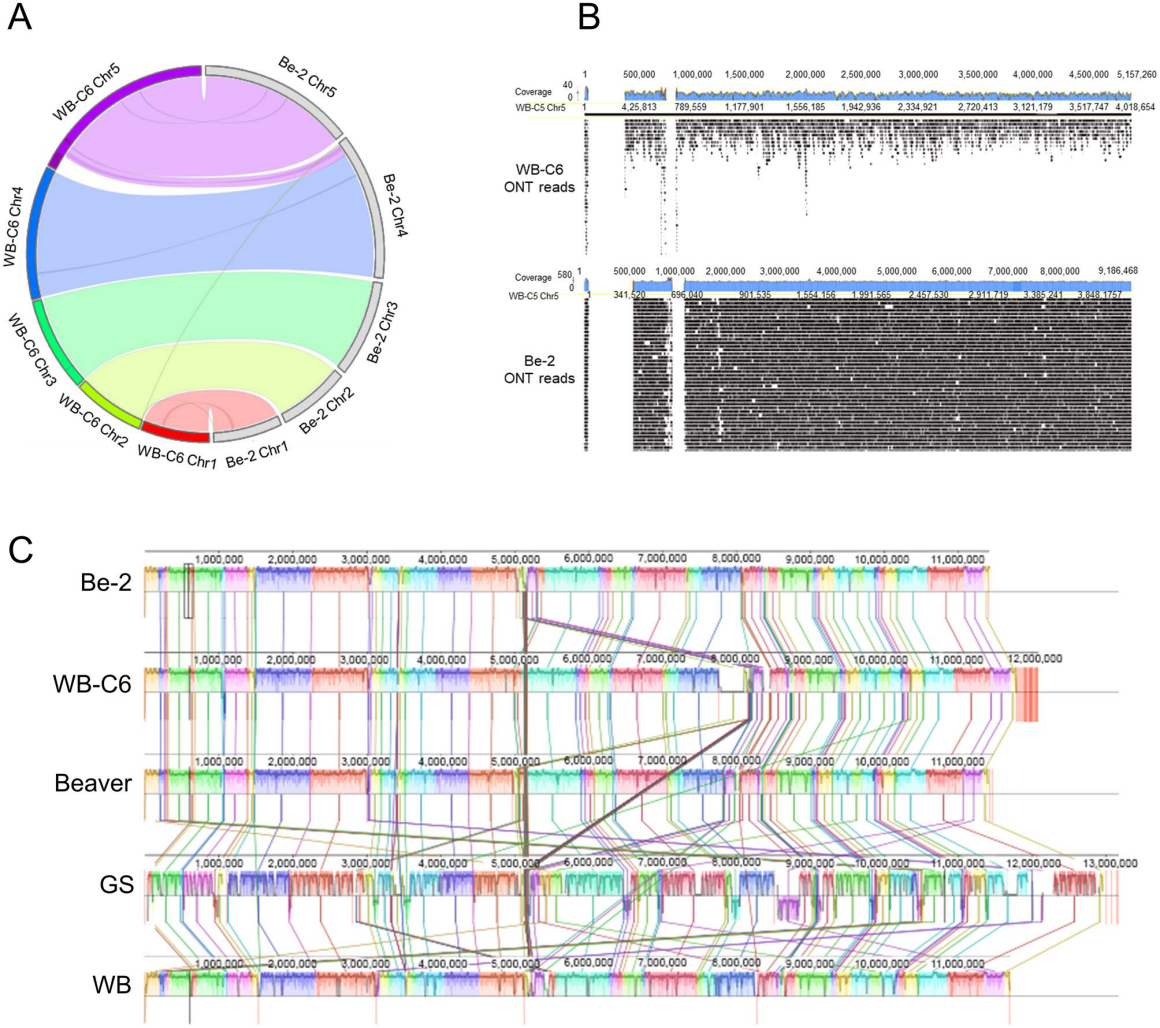
Synteny map of *G. duodenalis* AI isolates. (**A**) Circular plot comparing the levels of synteny among the *Giardia* strains Be-2 (right) and WB-C6 (left)^24^. The outermost grey circle shows the chromosomal organization of the Be-2 genome and compares with annotated chromosomes of WB-C6^24^ with colored bands and lines linking connected syntenic blocks. The dark pink color in WB-C6 Chr5 represents the gap in the assembly of the reference genome of *G. intestinalis* WB-C6^24^. Each chromosome is labeled with a strain name followed by chromosome number. The Circos plot was drawn with R library circlize^55^. (**B**) Long read alignment at Chr5 of *G. intestinalis* WB. White spaces at the five-prime region of the chr5 represent gaps with no physical evidence from long reads. Blue bar plots indicate coverage of ONT long-reads across the genome. Chromosomes are scaled in kilobase pairs. (**C**) Multiple whole-genome alignments were constructed using the Mauve plot^37^ of five long-read assembled *Giardia* genomes^24,35^. Each horizontal line with homologous segments outlined as colored rectangles represents each genome separately. Inverted segments are set below those that match the forward orientation. Each connecting line represents the aligned blocks between genomes. White areas possibly contain genome-specific sequence elements and those genomic positions that did not adequately align between the selected genomes.

By aligning the Be-2 assembled sequences to the published WB-C6 reference genome^24^, we observed a high synteny (Fig. 2A). However, we also noticed that a distal portion of Chr5 of WB-C6 genome instead corresponds to the Chr4 of the *G. duodenalis* Be-2 genome (Fig. 2A). Since this region was filled with gaps in the reference WB-C6 genome^24^, we checked for long read support to validate if there was any physical support for the previous assembly. After comparing WB-C6 PacBio and Be-2 ONT long-reads against the original WB-C6 genome, we were able to determine no physical evidence to fuse these contigs into Chr5 (Fig. 2B, Supplemental Fig. S1). Additionally, the depth plot revealed the presence of two gaps in Chr5 of WB-C6, and the small gap region serves as the breaking point. However, there are no gaps in Chr4 of Be-2, which indicates that the long-reads are bridging the gap region in Chr4 of Be-2 (Supplemental Fig. S1). Hence, the depth plots from Chr4 in the *G. duodenalis* Be-2 genome showed good support that those regions are indeed at Chr4 (Fig. 1, Supplemental Fig. S1), suggesting that the published WB-C6 assembly was incorrect, mistakenly assembled to the five-prime region of Chr5 of WB-C6.

### Comparing the Be-2 genome with other recently published long-read assemblies of *Giardia*

Long-read sequencing elucidates structural variation and evolution in protozoan parasites. Recently, Pollo et al.^35^ conducted a de novo hybrid assembly of *G. Intestinalis* Assemblage A isolate WB, Assemblage B isolate GS, and an isolate from a beaver strain using Oxford Nanopore long-read and Illumina short-read sequences. These data provide us with an opportunity to conduct comparative genome analysis and understand the completeness of the Be-2 genome. Our assembly of Be-2 is more contiguous than the previously published WB, GS, and Beaver genomes; it has fewer contigs (9, compared to 37, 19, and 8 contigs for WB, GS, and Beaver respectively) and a higher contig N50 value (3,045,186, compared to 616,181, 1,645,020, and 1,964,742 for WB, GS, and Beaver isolate respectively) as shown in Table 1^24,35^. These genomes share a high degree of synteny as represented in a Mauve plot^37^ (Fig. 2C), particularly between the Beaver and WB genomes. Mauve alignment also identified multiple inversions in the GS strain, with an assembly implying a much bigger genome (~ 13 Mb for GS to ~ 11 Mb for Be-2). Additionally, we identified misalignment in the WB-C6 reference genome in the 5′ region of Chr5 compared to all four strains (Be-2, Beaver, GS, and WB).

### Phylogenetic analysis

**Figure 3:**
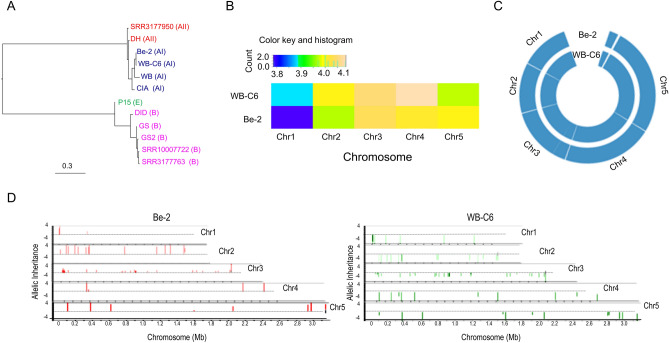
Comparative genomics of *G. duodenalis* AI isolates. (**A**) Maximum likelihood phylogenetic tree was conducted using all core accessory genes annotated in all analyzed isolates. The upper clade represents the assemblage A isolates, whereas the bottom cluster includes the assemblage B and E isolates. Assemblages are shown in brackets on the basis of previous designation. Assemblage (**B**) Comparative analysis of somy between Be-2 and WB-C6 indicates the 4N genome^24^. Somies were calculated based on the average of block coverages which is scaled to the ploidy of the strains using AGELESS software (http://ageless.sourceforge.net/) in a rolling window of 2000 bp and averaging the coverage within each window^69^. Somies were plotted using ggplot in R packages (v.4.1.0, URL: http://www.R-project.org). (**C**) Genome-wide zygosity profiles in *Giardia* strains Be-2 and WB-C6 identified long stretches of homozygosity. The total number of SNPs was calculated using a rolling window of 2000 bp. The blue, red, and yellow colors represent the homozygous SNPs (> 90%), heterozygous SNPs (> 90%), and both homozygous and heterozygous SNPs (50% each) respectively. (**D**) Bottlebrush plots showing the allelic inheritance pattern and loss of heterozygosity in the assemblage A isolates Be-2 and WB-C6. The X-axis represents the size of chromosomes (Mb), and the Y-axis shows allelic inheritance. Red and Green bars represent allele frequencies of Be-2 and WB-C6 respectively.

We employed phylogenetic reconstruction to confirm the assemblage type for Be-2 by downloading all genome assemblies comprising fewer than 1000 fragments and bearing an assemblage designation, emphasizing those from assemblages AI, AII, and B. Additionally, we examined whether including fragmented assemblies derived from short-read data affected phylogenetic inferences. Doing so placed sub-assemblages AI and AII in a single node, distinct from highly diverse clusters of assemblages B and E (Fig. 3A). Our assembled strain, Be-2, clustered very closely with WB-C6, confirming their membership in sub-assemblage AI strains (Fig. 3A).

### G+C content comparison

The G+C content of Be-2 is 49.58%, comparable to that reported for the WB strain^23^. However, the recently published WB-C6 genome estimated a G+C content (46.3%)^24^ lower than our estimate and lower than strains DH_A2 (49.04%), and GS_B (48.25%)^36^. Low estimates of G+C content for the P15_E (46%), and GS_B (47%) may derive from a failure to assemble G+C rich repeat regions^25^. Thus, variable estimates of G+C content likely derive from true variation in genome content and methodological limitations.

### Genome ploidy in the vegetative trophozoite stage

To estimate the nuclear genome ploidy of Be-2 during the vegetative trophozoite stage, we calculated the number of reads aligning to each chromosome. We estimated the number of copies (somies) of each chromosome by averaging coverage in a rolling window of 2000 bp^38^. Doing so established tetraploid nuclei (4N) for trophozoites of WB-C6 and Be-2 (Fig. 3B); we encountered no evidence of large structural duplications or aneuploidy, which varies between 3.8 and 4.1 per chromosome based on the heatmap scale (Fig. 3B), and corresponds exactly with the published article by Tůmová et al.^39^. Thus, neither strain undergoes a haploid stage during the vegetative cell cycle^26^.

### Allelic sequence heterozygosity estimation

*G. duodenalis* and related diplomonads possess two nearly identical, transcriptionally active nuclei. Low heterozygosity characterizes parasites in assemblage A^24,36^. We estimated a low rate of heterozygosity (0.0117%; Fig. 3C) in the nearly complete genomes of WB-C6 and Be-2, confirming prior estimates of related lineages^24,36^.

### Allelic inheritance pattern and frequency

To understand the pattern and the distribution of genetic differences between two transcriptionally active nuclei, we developed a high-resolution map of single nucleotide differences to depict the inheritance pattern and allele frequencies throughout five chromosomes of *Giardia* using a bottle brush plot (Fig. 3D). The bottle brush plots were developed by mapping Illumina short-reads against the chromosomal-scale reference genomes of Be-2 and WB-C6, as described in the methods section. These plots showed a near-complete loss of heterozygosity in both Be-2 and WB-C6 genomes with the presence of very few mutations (Fig. 3D). Comparative analysis of the inheritance pattern and allele frequency of these mutations indicates the spontaneous accumulation of mutation and no recombination in Be-2 and WB-C6 genomes (Fig. 3D), indicating the presence of two genetically identical diploid nuclei in both Be-2 and WB-C6.

### BUSCO analysis for genome completeness

After conducting the de novo assembly using Nanopore long-reads and polishing with well-covered Illumina short-reads (99.86%), we used Benchmarking Universal Single-Copy Orthologs (BUSCO) to evaluate quantitative measures of the genome assembly using evolutionarily related gene content from near-universal single-copy orthologs^40^. We used Eukaryote datasets (Superkingdom: eukaryota_ odb10), which contain 255 single-copy orthologs. The BUSCO assessment of Be-2 yielded a completeness score of 24.3% (23.5% completed, 0.8% duplicated), and 5.5% were fragmented.

### Annotation and comparative genomics of *G. duodenalis* Be-2

**Figure 4:**
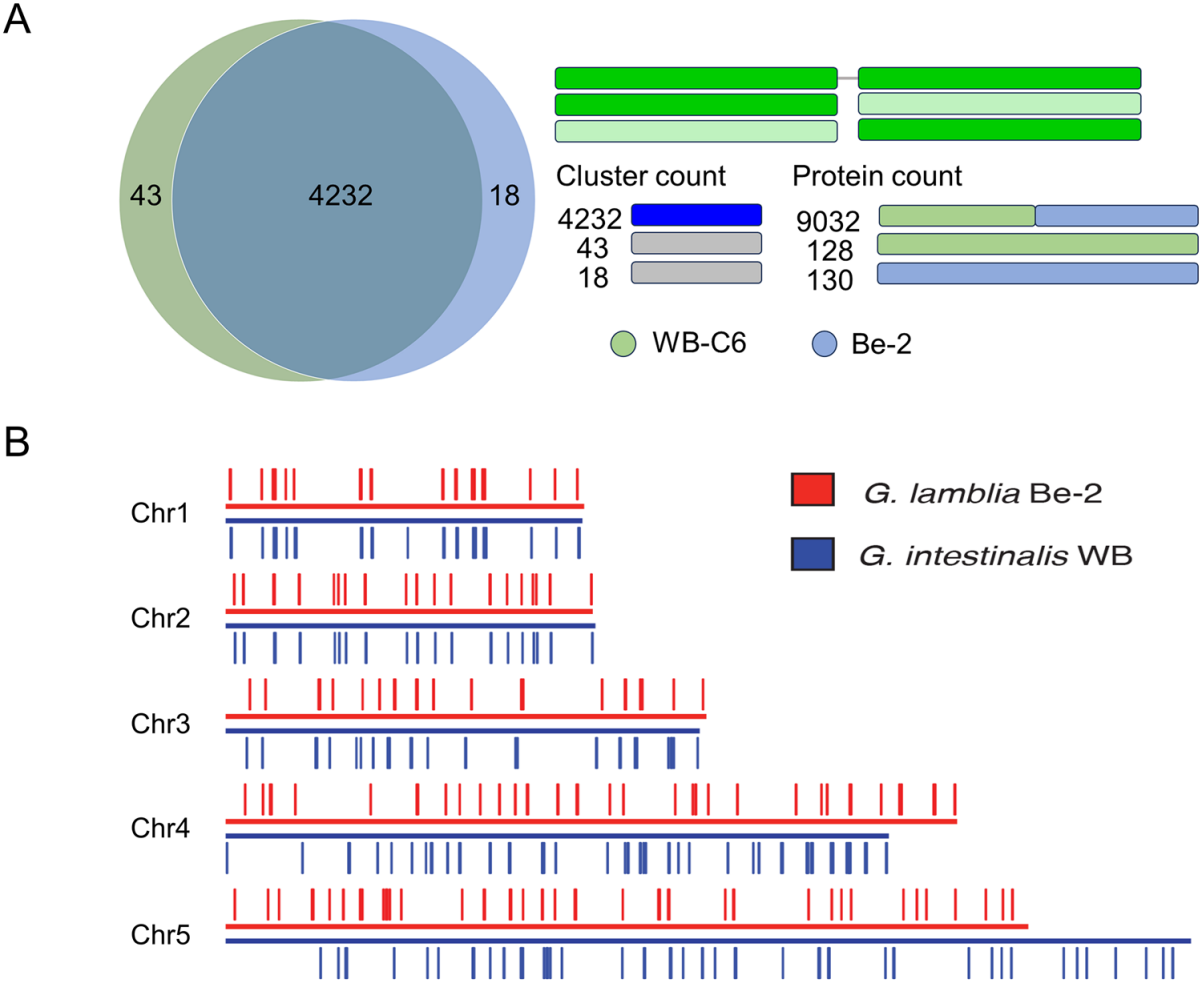
(**A**) The Venn diagram illustrates the number of shared and specific genes between Be-2 and WB-C6 based on clusters of orthologs. Horizontal bar plots illustrate the comparative analysis of protein sequences on the 43 and 18 orthogroup clusters representing WB-C6 and Be-2 unique orthogroups. The green color represents WB-C6 whereas the blue color represents Be-2. (**B**) VSP gene distribution on the assembled genome of Be-2 and WB. Red lines represent the VSPs present in Be-2 whereas blue lines indicate VSPs in WB. Chromosomes are scaled in kilobase pairs.

To determine similarities and differences between Be-2 and WB-C6, we analyzed their annotated orthologous clusters using OrthoVenn and inferred phylogenetic relationships between the two strains (Fig. 4A). We found that Be-2 and WB-C6 have a high degree of shared orthologs (4232 genes), similar to previously published data (supporting 4,557 orthogroups) (Fig. 4A). We identified 18 genes unique to Be-2 and 43 genes unique to WB-C6. Interestingly, the number of genes unique to WB-C6 grows (to 337 orthologues) when accounting for singletons (Fig. 4A). The more fragmented nature of the WB-C6 assembly or differences in the annotation methods may contribute. Comparing protein sequences on the 43 and 18 orthogroup clusters representing WB-C6 and Be-2 unique orthogroups respectively, (Fig. 4A) revealed about 130 proteins in each group with very similar functions, suggesting that the genome of WB-C6 is more fragmented than that of Be-2.

*G. duodenalis* genomes encode large families of cysteine-rich proteins, which include variant-specific surface proteins (VSPs) that act as virulence determinants in *Giardia*^41^. VSPs contain many CXXC motifs, one or two GGCY motifs, a conserved hydrophobic tail, and a Zn finger motif; their diversity drives antigenic variation in *Giardia*^42^. Including VSPs as vaccine antigens enhances protection against *Giardia* in dogs and rodents^43^.

With one notable exception, we noted a high degree of synteny between the Be-2 and WB-C6 strains when comparing the position of VSPs across their chromosomes (Fig. 4B). Rearranging the 5′ region of Chr5 in the published assembly for WB-C6 genome (Fig. 2) resolves this notable exception.

## Discussion

Until the advent of long-read sequencing technology, attempts to sequence *Giardia* (which began in 2007^23^) employed Sanger sequencing and then Illumina and 454 short-read technologies^23–25,30^. These methods produced highly fragmented genomes with scaffold numbers ranging from 200 to 800. We employed the long-read Nanopore platform with Illumina polishing to achieve an improved assembly of *Giardia duodenalis* Sub-assemblage A1 (Strain Be-2), condensed to only 9 contigs. Comparative analysis identified one significant conflict with the recent reference genome of WB-C6. Strictly, comparative genomics showed remarkable homozygosity and sequence similarity of the two nuclei within a cell and the conservation of VSP genes among Sub-assemblage A1.

Despite recent efforts to sequence genomes of *Giardia,* the extent of intra-sub-assemblage genetic diversity remains poorly defined^30^, impeding efforts to understand disease etiology, transmission mode, and evolution through genetic exchange. Whole genome sequencing employing long read approaches improves de novo assembly, mapping, transcript isoform identification, and structural variant detection. The 2007 WB genome sequence served as a reference until a recent effort, using the PacBio platform combined with optical mapping, provided a nearly complete chromosomal assembly for the WB-C6 isolate in sub-assemblage AI of *G. duodenalis*^24^. The WB-C6 genome reduced the contigs for the WB strain from 306 to 38 and reduced the number of gaps from 137 to 4^23,24^. Our assembly of the Be-2 genome using long-read Nanopore sequencing yields five complete chromosomes spanning 11,432,336 bp with only one internal gap. Our assembly of Be-2 places a region previously ascribed to the 5′ region of Chr5 in the reference genome WB-C6 instead as the three-prime region of Chr4, which contains approximately four copies of non-LTR LINE-like retrotransposon GilM^24^. We resolved this conflict as a misassembly artifact (rather than a true difference in genome structure) by employing other genomes assembled from long reads (including WB, GS, and another Beaver isolate^35^). Our methods located that missing piece at the 3-prime region of Chr4, providing greater accuracy; only one small gap persists, in a repeat-laden subtelomeric region of Chr3. Our more complete and better annotated Be-2 genome serves as a resource for population and comparative genomics studies seeking to understand host specificity, virulence, and transmission.

BUSCO analysis suggests our Be-2 genome assembly is more complete than other published genomes for *Giardia,* paralleling completeness reported for other eukaryotic genomes. Our assembly includes only 0.8% duplications, which may include contributions from multigene families. Hence, the current nearly-completed genome of assemblage AI Be-2 should prove a valuable tool for future comparative genomic studies to understand the molecular basis of transmission and differential manifestation of disease outcomes.

The early-diverging enigmatic protist *G. duodenalis* is unusual in that it harbors two identical, transcriptionally active diploid nuclei in the vegetative trophozoite stage^26^. We identified a tetraploid (4N) genome in each chromosome of WB-C6 and Be-2 trophozoites. The paired nuclei appear notably lacking in genetic diversity and heterozygosity in *G. duodenalis* belonging to the AI sub-assemblage. How they maintain such homozygosity and identical nuclei deserves further investigation. Neither mating nor meiosis has been reported in *Giardia*. If *Giardia* does not go through a sexual cycle, then each nucleolus should accumulate spontaneous mutations, leading to the generation of a heterozygous genome. In contrast, sexual reproduction in eukaryotic organisms allows for reassortment and removal of deleterious mutations, producing better-fit genomes^44^ Ramesh et al. identified and verified the presence of a core set of putative meiotic genes, including five meiosis-specific genes, indicating that *Giardia* is capable of sexual reproduction^45^. Although there is no evidence of karyogamy and exchange of genetic material in trophozoites, Poxleitner et al., showed the fusion of nuclei and somatic homologous recombination in cysts^46^. Additionally, genetic exchange without meiosis has also been observed in *Giardia*, promoting the hypothesis that reduced heterozygosity results from cyst nuclei exchange of genetic material rather than nuclear sorting. Hence, there is ample evidence that *Giardia* does sexually recombine, and further research is needed to determine whether it involves meiosis or a parasexual cycle. Assembling different assemblages of *Giardia* with long-read sequences could provide a basis to study the role of sexual recombination in *Giardia* genome evolution and the forces shaping population genetic structure^47^.

Each assemblage of *Giardia* possesses unique virulent factors, and hosts mount a range of responses to parasites of a given sub-assemblage. Interestingly, transcriptomic and proteomic analyses have identified glycolytic and arginolytic enzymes, cysteine proteases, and VSPs as potential factors for prolonged infection and virulence^43^. Although previous studies identified an estimated repertoire of 270–303 VSP gene sequences comprising 4% of the entire genome, *Giardia* trophozoites express only a single member of this protein family at a time by switching the expression every 6–13 generations from one VSP to another, causing antigenic variation in hosts^48^. Interestingly, in other eukaryotic pathogens like *Plasmodium* and African *Trypanosome*s, variant surface antigen genes (var genes for *Plasmodium* and VSGs for *Trypanosoma*) are frequently located in sub-telomeric regions; however, sub-telomeric location of VSP genes is relatively uncommon^48^. Our comparative analysis of VSP genes of *Giardia duodenalis* Sub-assemblage A1 strains Be-2 and WB-C6 also showed the widespread distribution of VSP genes throughout chromosomes. Additionally, we found striking syntenic conservation in the genomic distribution of VSPs in Sub-assemblage A1 when comparing WB-C6 to Be-2. Such conservation may attest to their importance to parasite reproductive success and host adaptation.

In conclusion, our approach achieves high-quality, telomere-to-telomere genomes. We acknowledged the importance of conducting comparative genomics with different assemblages of *Giardia* with nearly completed reference genomes with regards to the VSP. Unfortunately, most existing whole genome sequences available for assemblages other than Assemblage A were assembled from short-read Illumina sequences that fail to bridge the repetitive genome regions. Our assembly, from long reads, produced less fragmentation. By lowering the cost and improving the quality of genome sequencing and assembly, this approach can support future, large-scale comparative genomic studies. High-resolution genetic maps may thereby enhance efforts to understand parasite diversity and evolution, elucidating the contribution of hybridization to population genetic structure and speciation, and opening new avenues for drug and vaccine development.

## Methods

### Parasite isolates and culture conditions

The Be-2 isolate (Beaver-2, IP-0583:1) was obtained from Biodefense and Emerging Infections Research Resources Repository (https://www.beiresources.org/Catalog/BEIParasiticProtozoa/NR-9238.aspx). Originally, Be-2 cysts were collected from the colon and rectum of a beaver in Canada in 1981. Cysts were fed to metronidazole-treated Mongolian gerbils to expand them; they were then axenized at the National Institute of Health (https://www.nih.gov). Be-2 was grown in ATCC Medium 2695 (Keister’s modified TYI-S33 medium) in tightly capped slanted culture tubes at 37 °C as described previously^49^. Trophozoites were harvested in the late log or early stationary phase after washing in phosphate-buffered saline solution.

### Preparation of gDNA and construction of the library for Nanopore sequencing

Total genomic DNA for whole genome sequencing was prepared from purified trophozoites using a DNeasy Blood and Tissue kit (Qiagen, USA) using approximately 10^8^ trophozoites according to the manufacturer’s instructions. After extraction, gDNA was purified again using MagBind TotalPure NGS beads (Omega BIOTEK, GA USA). Agilent 4150 TapeStation system (Agilent, CA, USA) was used to check the integrity and size distribution of the gDNA using the Genomic DNA reagent kit. Total gDNA concentration was calculated using a Qubit 4 Fluorometer (Invitrogen, ThermoFisher Scientific, USA). The ratios of absorbance at 260 and 280 nm, and 260 and 230 nm were calculated to assess the DNA purity by spectrophotometric analysis using Nanodrop (ThermoFisher Scientific, USA). 400 ng of high-quality and high molecular weight DNA was utilized to prepare genomic libraries using the SQK-RAD004 rapid sequencing kit (Oxford Nanopore Technologies ONT, UK) according to manufacturer instructions. Nanopore sequencing was conducted for 24 h on a MinION R9.4.1 flow-cell (FLO-MIN106D) using ONT protocols.

### Basecalling and primary data analysis for long-read Nanopore sequencing

Primary data acquisition was conducted by MinKNOW software (Oxford Nanopore Technologies ONT, UK) to produce FAST5 (HDF5) files and FASTQ files. FAST5 files were utilized for basecalling using Oxford Nanopore Technologies Guppy V.6.1.2^50^ software with average quality scores > 7. High-quality basecalled reads were further processed with Porechop (v0.2.3) (https://porecamp-au.github.io/) to remove adapters and chimeric reads.

### Short-read Illumina sequencing

Libraries for whole genome sequencing were produced from *G. duodenalis* Be-2 strain using an Illumina DNA Prep kit (Illumina, San Diego, CA, USA). Approximately 200 ng of Be-2 gDNA was used as input for library preparation in triplicate for sequencing. Individual libraries were pooled together at the end of the procedure. Individual and pooled libraries were quantified by Qubit 4 Fluorometer (Invitrogen, ThermoFisher Scientific, USA), and the size distribution of libraries was characterized using a Bioanalyzer 2100 (Agilent, CA, USA) and an Agilent 4150 TapeStation system (Agilent, CA, USA). Library size profiles were within the manufacturer’s recommended range of 150–1500 bp and the average fragment for each library ranged 303–473-bp. The pooled library had an average fragment size of 420 bp. Libraries were sequenced with a MiSeq Reagent v2 kit (500 cycles) on an Illumina MiSeq system in a format of 250 × 2 paired-end reads. Reads were demultiplexed and files were exported in fastq format. High genome coverage (99.86%) Illumina short reads were generated to assist in base error correction of the long-read Nanopore sequencing reads.

### Whole genome assembly and annotation

The de novo assembly was performed using Flye v.2.9^51^ utilizing just nanopore-raw reads with the scaffolding parameter set as off. The resulting contigs were then polished using NexPolish v.1.4.1^52^ using Illumina reads with the following parameters in the configuration file: task = best; rerun = 3; genome_size = auto; sgs_options = -max_depth 100 -bwa. After the assembly all contigs were submitted to RagTag v.2.1.0^53^ using the WB strain genome as a reference, just to put all contigs in the same orientation as the reference genome. No scaffolding was performed at this point to avoid any structural bias. Scaffolding was done later based on Ragtag scaffolding results supported by the graphs generated from the Flye assembler (.gfa files).

Genome statistics were generated using QUAST v.5.2^54^ and plots were generated using the R library circlize^55^. GC% was calculated across the genome using GCcalc.py (https://github.com/WenchaoLin/GCcalc) with a 1 kb sliding window size.

The annotation was made in two steps. First, we used Liftoff v.1.6.3^56^ to transfer known annotated genes from WB-C6 to our assembly, using the flag -copies -sc 0.9 -infer_genes -infer_transcripts, to get any potential new paralog in our new assembly. Liftoff partial transfers were flagged and removed after final curation. Second, we trained an AUGUSTUS database^57^ using the WB annotation to perform an ab initio gene prediction, to potentially find new genes in the new genome assembly. Both gene prediction models were manually curated using Webapollo 2.0.7^58^. Bedtools intersect v.2.30^59^ was then used to check unique gene predictions from AUGUSTUS^57^ to be validated before adding to the final gff. rRNA genes were predicted using infernal v.1.1.4^60^. The final annotation was submitted to InterproScan v5.53^61^ to classify the annotated proteins by families and identify the functional domains that would validate the functional annotation transferred from Liftoff.

### Phylogenetic analysis

Since most of the publicly available genomes don’t have any genome annotation available, first we generated a genome annotation based on homology utilizing Liftoff^56^ using the same parameters as mentioned above to develop a phylogenetic tree. Additionally, for the genomes with available annotations, we decided to also use our method to maintain the prediction consistent among all samples compared. The annotation, containing the genome sequences was formatted to meet the criteria for the Roary pipeline^62^ to generate the MAFFT v7.508^63^ alignments. These alignments were submitted to Fasttree v2.1.11^64^ to reconstruct the ML tree.

### Comparative analysis

Synteny analysis between *G. duodenalis* Be-2 and WB was performed using minimap2 v.2.24^65^, and Progressive Mauve 2.4.0^37^ using default settings. And plotted in R using circlize^55^. Ortholog analysis between both genomes was made by Orthofinder v.2.5.4^66^ and the results were plotted using Venn webtool (https://bioinformatics.psb.ugent.be/webtools/Venn/).

### BUSCO analysis

Genome completeness and redundancy were estimated using BUSCO v5.4.2, which is in synchronize with the OrthoDB v10 release with genome mode and the eukaryotic lineage^40^.

### Variant calling using short-read Illumina data

The Illumina paired-end reads for each of the *Giardia* strains were mapped onto the current genome assembly of Be-2 using the Burrows-Wheeler Aligner (BWA, v2.2.1) mem^67^ in default parameters. Mapped reads were then converted to a bam file and sorted using SAMtools v1.6^68^. Quality score recalibration and variant calling were conducted using Genome Analysis ToolKit (GATK, v,4.2.0.0) with HaplotypeCaller with a read coverage ≥ 10X, -stand-call-conf 30.0, and -sample-ploidy 4.

### Somy calculation

Somies were calculated using AGELESS software (http://ageless.sourceforge.net/) in a rolling window of 2000 bp and averaging the coverage within each window^69^. Regions with greater than twice and less than half the average coverage and zero coverage for each chromosome were removed from the analysis. The somies of each chromosome were calculated based on the average of block coverages which is scaled to the ploidy of the strains. Somies were plotted using ggplot in R packages (v.4.1.0, URL: http://www.R-project.org).

### Homozygosity and heterozygosity calculation

We compared the Be-2 strain to the WB strain to identify variant positions. We designated SNPs as either heterozygous (in one or both strains) or homozygous (consistently distinguishing the two strains) and estimated heterozygosity rates in 2000 bp rolling windows using custom Java scripts to determine histogram plots in Circos^70^. The presence of 90% or more heterozygous SNPs was depicted by red color whereas the presence of 90% or more homozygous SNPs was indicated by blue color.

### Chromosomal inheritance patterns

Chromosomal inheritance patterns were determined using AGELESS software by determining the allele composition of each SNP^69^. The allele composition at loci with coverage greater than or equal to 10, and allele frequencies between 0.15 and 1.0 were considered. Allele composition per strain was plotted using a bottle brush plot as described previously^69^.

### Supplementary Information

Supplementary Figure 1.

## Data Availability

Raw Nanopore long-read genomic sequences and whole genome short-read Illumina sequences were deposited in the NCBI Sequence Read Archive SRA page under the following BioProject ID: PRJNA901457 (URL: http://www.ncbi.nlm.nih.gov/bioproject/901457; accessions are CP110916-CP110920).
