# Supplementary material for: Comparative genomics of Giardia duodenalis sub-assemblage AI beaver (Be-2) and human (WB-C6) strains show remarkable homozygosity, sequence similarity, and conservation of VSP genes
Source: Sci Rep. 2024 Jun 12;14:13582. doi: 10.1038/s41598-024-63783-5 (PMC11169602; doi:10.1038/s41598-024-63783-5)
Supplement: Supplementary file 1 — Supplementary Figure 1. [file 41598_2024_63783_MOESM1_ESM.pdf]

Supplementary material

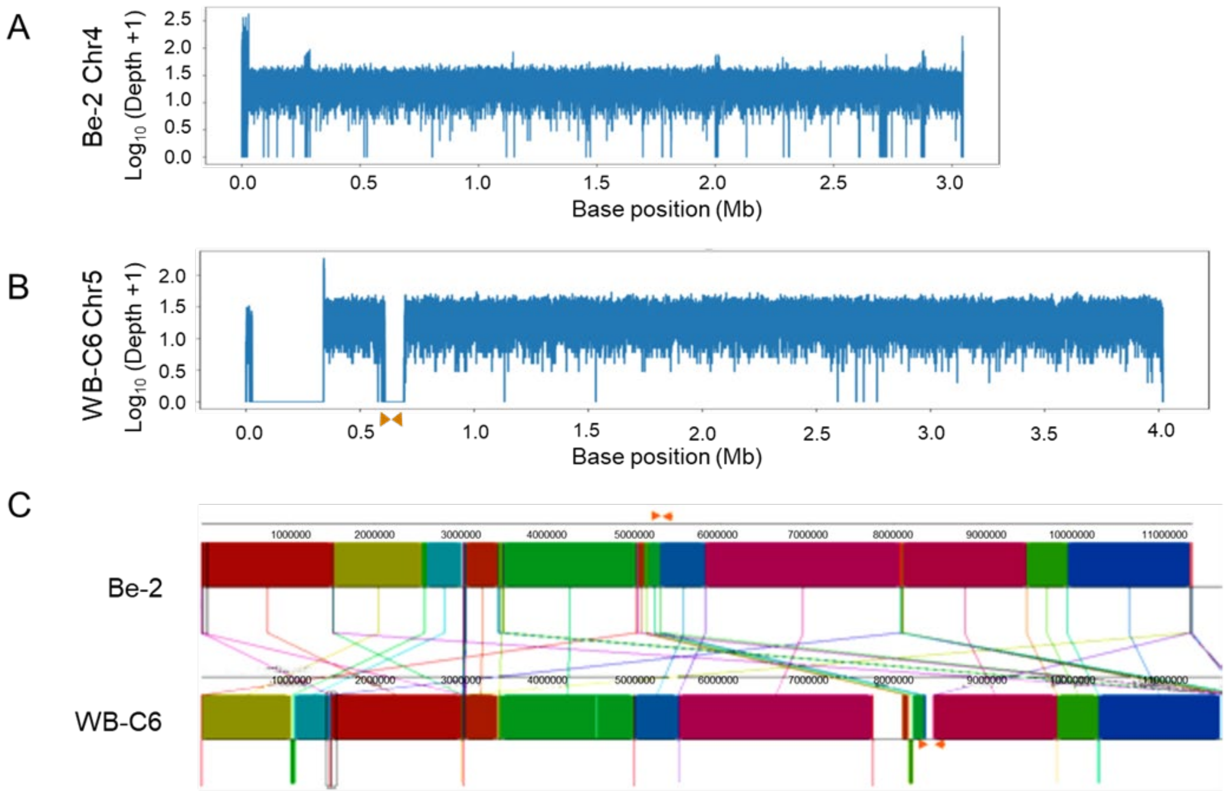

Supplemental Fig. S1. Long-read coverage plots revealed no gaps in Chr4 of Be-2. A) Long-read coverage plot of Be-2 Chr4 showed no gaps. The Y-axis indicates the number of mapped long reads, while the X-axis indicates the base position of the chromosome. B) Long-read coverage plot of WB-C6 Chr5[24] showed two gaps in Chr5. Arrowheads indicate the breakpoint. The Y-axis indicates the number of mapped long reads, while the X-axis indicates the base position of the chromosome. C) Pairwise whole-genome alignments using the Mauve plot[37] showed the location of the breakpoint and the gaps in Chr5 of WB-C6, which are absent in Be-2 Chr4. Arrowheads indicate the breakpoint.
